# Microcystin Biosynthesis and* mcyA* Expression in Geographically Distinct* Microcystis* Strains under Different Nitrogen, Phosphorus, and Boron Regimes

**DOI:** 10.1155/2016/5985987

**Published:** 2016-10-10

**Authors:** Ankita Srivastava, So-Ra Ko, Chi-Yong Ahn, Hee-Mock Oh, Alok Kumar Ravi, Ravi Kumar Asthana

**Affiliations:** ^1^Cell Factory Research Center, Korea Research Institute of Bioscience & Biotechnology, Daejeon, Republic of Korea; ^2^Ocular Biochemistry, Dr. R. P. Centre for Ophthalmic Sciences, All India Institute of Medical Sciences, New Delhi 110029, India; ^3^Centre of Advanced Study in Botany, Banaras Hindu University, Varanasi 221 005, India

## Abstract

Roles of nutrients and other environmental variables in development of cyanobacterial bloom and its toxicity are complex and not well understood. We have monitored the photoautotrophic growth, total microcystin concentration, and microcystins synthetase gene (*mcyA*) expression in lab-grown strains of* Microcystis* NIES 843 (reference strain), KW (Wangsong Reservoir, South Korea), and Durgakund (Varanasi, India) under different nutrient regimes (nitrogen, phosphorus, and boron). Higher level of nitrogen and boron resulted in increased growth (avg. 5 and 6.5 Chl *a* mg/L, resp.), total microcystin concentrations (avg. 1.185 and 7.153 mg/L, resp.), and* mcyA* transcript but its expression was not directly correlated with total microcystin concentrations in the target strains. Interestingly, Durgakund strain had much lower microcystin content and lacked microcystin-YR variant over NIES 843 and KW. It is inferred that microcystin concentration and its variants are strain specific. We have also examined the heterotrophic bacteria associated with cyanobacterial bloom in Durgakund Pond and Wangsong Reservoir which were found to be enriched in Alpha-, Beta-, and Gammaproteobacteria and that could influence the bloom dynamics.

## 1. Introduction

Bloom-forming freshwater cyanobacterial genera such as* Microcystis*,* Oscillatoria*,* Anabaena*, and* Nostoc* produce toxins and other bioactive compounds that can poison and kill humans and livestock [1, 2].* Microcystis* sp. is the most frequently encountered one in freshwater bodies and is associated with production of hepatotoxic microcystin (MC) [3]. MCs are coded by the microcystins synthetase (*mcy*) gene cluster having 10 genes [4]. The gene cluster is transcribed in two polycistronic transcripts (*mcyABC* and* mcyDEFGHIJ*). The larger,* mcyD-J*, encodes a modular polyketide synthase (PKS) (*mcyD*), two hybrid enzymes comprising nonribosomal peptide synthetase (NRPS) and PKS modules (*mcyE* and* mcyG*), and enzymes putatively involved in the tailoring (*mcyJ*,* F*, and* I*) and transport (*mcyH*) of the toxins. The smaller operon,* mcyA-C*, encodes three NRPS enzymes. More than 89 MC variants have been described from natural blooms and laboratory cultures of cyanobacteria [2]. The physiological role of MCs in the producing organism is little understood. However, their roles were proposed in extracellular signaling and autoinduction [5, 6], photosynthesis [7], protein modulation [8], scavenging of oxygen radicals [9], and maintenance of colonies [10].

Parameters like light intensity, pH, temperature, nutrients, and trace metals could play a critical role in MCs production [11, 12]. Various field studies have demonstrated that nutrient enrichment of aquatic bodies by nitrogen (N) and/or phosphorus (P) could promote toxic blooms of* Microcystis* [13, 14]. In large aquatic systems highly complex interactions among the physical, chemical, and biological variables regulate the proliferation of* Microcystis* blooms and toxin production, which could lead to contradicting results [13]. Laboratory studies have also shown the effects of nutrients on the growth and MC production of* Microcystis* [15, 16]. Only a few studies have focused on response of* Microcystis* to differing nutrient concentrations and trace metals on* mcy* transcription and MCs production [17, 18]. Recently, bacterial population associated with* Microcystis* bloom also received attention for its role in development of bloom [19]. A potential role of boron (B) in signaling and bacterial interspecies communication [20] aroused interest in studying its role in growth of* Microcystis* sp. and MC production in lab cultures, in addition to N and P.

A part of gene* mcyA* codes for the section of the condensation domain that contains the conserved motif C5 and is essentially required for MC biosynthesis in* Microcystis* sp. [4]. Therefore, we have monitored the* mcyA* gene expression and MC production and growth of three different strains of* Microcystis*, namely,* M. aeruginosa* NIES 843 (reference strain),* M. aeruginosa* KW (Wangsong Reservoir, South Korea), and* Microcystis* sp. (local pond, Durgakund, India), under selected nutrient regimes. As we had accessibility to natural algal bloom material from Durgakund Pond and Wangsong Reservoir, a seasonal variation in community dynamics of bacteria associated with cyanobacteria was also investigated.

## 2. Materials and Methods

### 2.1. Photoautotrophic Growth of Target Strains of* Microcystis* sp. and MC Analysis

Culture of* M. aeruginosa* NIES 843 strain and* M. aeruginosa* KW strain was maintained in BG-11 medium [21].* M. aeruginosa* Durgakund strain was isolated from bloom samples collected from Durgakund Pond, Varanasi, India (25°17′20′′N, 82°59′58′′E), in which cyanobacterial blooms were noticed throughout the year. Our previous study characterized the real state of cyanobacterial bloom composition and the levels of MC variants in the target pond [12]. This pond lies 8.77 m above the sea level and has an area of 8010 m^2^ with a mean depth 26.6 m. The pond is not connected to any river with exception of incoming water from adjacent temples and is often used for various religious activities. Samples were purified by repetitive subculturing in solid (soft agarose) and liquid culture media alternately [22]. The strains were grown in 165 mL sterilized BG-11 in 250 mL flasks. The cultures in all of the experiments were incubated at 25 ± 0.5°C with cool white fluorescent lights (80 *μ*mol photons m^−2^ s^−1^, 18 h light/6 h dark).

Growth and MC production curves of all three strains were determined under different nutrient (N, P, and B) regimes. Nutrients levels were selected based on the concentrations in BG-11 medium (comparatively nutrient rich media) and further decreased according to N/P ratio in cells [23].* Microcystis* strains were individually grown in 250 mL culture flasks for 15 days in sterile BG-11 medium under low nitrate (0.015 mM), low phosphate (0.001 mM), and low boron (0.23 *μ*M) separately. The growth of the cultures was followed up every alternate day. Samples were filtered (GF/C, Whatman, UK) and suspended in 80% acetone for overnight in dark (4°C). The supernatant was used to measure chlorophyll* a* (Chl* a*) (665 nm) according to Myers and Kratz [24]. A known volume of samples was collected four times (days 1, 3, 5, and 11) during the study period, filtered (GF/C, Whatman, UK), and stored until use for total (intracellular and extracellular) MC analysis. MC data are expressed as total MC concentration and as a ratio to Chl *a* to indicate the change in MC content. Extraction and estimation of three MC variants (MC-LR, -RR, and -YR) were carried out as previously described [12]. After starvation, 15 mL culture was washed and transferred separately to 250 mL flasks containing 150 mL sterilized BG-11 with higher N concentrations (1.5 mM and 17.6 mM) while P and B concentrations were kept constant as those of standard BG-11 medium. The experiment design was the same for all the three strains for higher P (0.1 mM and 0.23 mM) and B (23 *μ*M and 46 *μ*M) levels.

### 2.2. RNA Extraction, cDNA Synthesis, and Real-Time RT-PCR

*Microcystis* cultures (15 mL) were harvested on 11th day (exponential phase) by centrifugation at 6,000 ×g for 10 min, and the cell pellets were resuspended in 1 mL TRI Reagent® (Sigma-Aldrich, USA). Zirconia beads (0.5 g, 0.2 mm; Biospec, Bartlesville, OK, USA) were added to the cell suspension and cells were disrupted by vortex for 60 s. Total RNA was isolated according to the manufacturer's instructions for the reagent and resuspended in 30 *μ*L of DEPC-H_2_O. RNA integrity was verified by agarose electrophoresis with ethidium bromide staining. The quantity and quality of RNA were assessed with a NanoDrop ND-1000 Spectrophotometer (NanoDrop Technologies, Inc.). For construction of cDNA, total RNA was digested using DNase I (New England BioLabs, MA, USA) to remove genomic DNA according to the manufacturer's instructions. The RNA was reverse transcribed into cDNA in 20 *μ*L reactions using iScript*™* cDNA synthesis kit (Bio-Rad, Hercules, CA) according to the manufacturer's instructions. Negative controls containing no reverse transcriptase were run simultaneously.

Real-time RT-PCR was carried out using CFX 96 C 1000*™* Thermal cycler (Bio-Rad, Hercules, CA). The reaction mixture consisted of 10 *μ*L of SsoFast EvaGreen Supermix, 1 *μ*L of each primer set for* mcyA* and 16S rRNA (Table 1), 1 *μ*L 1 : 5 dilution of cDNA, and 7 *μ*L of sterile Milli-Q-H_2_O. Negative controls (without template DNA) were run simultaneously. The quantitative PCR program consisted of 94°C (5 min) and 40 cycles of 94°C (30 s), 60°C (30 s), and 72°C (30 s) with a final extension of 72°C (5 min). The annealing temperature for 16S rRNA gene was increased to 62°C. The fold change in the expression of the target gene relative to the control cells (grown in low nutrient concentrations) was calculated using CFX Manager Software (Version 2.1) and normalized with the expression of 16S rRNA (reference gene) [30].

### 2.3. Field Samples

Water samples were collected in 2 L acid washed glass bottles (Durgakund Pond) and 20 L polyethylene bottles (Wangsong Reservoir) from surface above a depth of 20 cm after some mixing and stored immediately at 4°C until the laboratory analysis was done. The samples were collected monthly from May 2010 to April 2011 and July 2010 to November 2010 during the cyanobacterial bloom only from Durgakund Pond and Wangsong Reservoir, respectively. The samples were filtered (0.2 *μ*m cellulose nitrate filter, Sartorius, Germany) to harvest most of the bacteria on the same day of collection and stored (−20°C) until use for molecular analyses.

### 2.4. DNA Extraction, PCR, and DGGE

DNA was extracted from the filter by grinding in liquid nitrogen and suspending in TE buffer (pH 8.0, 10 mM Tris-HCl and 1 mM EDTA) followed by phenol/chloroform method [31]. A nested-PCR was carried out for the amplification of nearly complete sequence of 16S rRNA with primers 27F and 1525R (Table 1). PCR was performed in a 50 *μ*L final volume of reaction mixture with 5 *μ*L of 10× buffer, 5 *μ*L of 2.5 mM dNTP mixture, 1.5 *μ*L of the respective primer sets (10 pmol), 1 *μ*L of template DNA, and 5 U of Ex-Taq DNA polymerase (Takara, Japan). The PCR protocol consisted of initial denaturation at 94°C (5 min) and 30 cycles of 94°C (30 s), 56°C (30 s), and 72°C (45 s) with a final extension of 72°C (10 min). This was followed by a touchdown PCR [32] with primers 341F (with 40 bp long-GC-clamp) and 907R (Table 1). The PCR protocol consisted of initial denaturation at 94°C (5 min) and 30 cycles of 94°C (45 s) and 72°C (45 s) with annealing temperature set to 65°C and decreased by 0.5°C at every cycle for 20 cycles, and then 15 additional cycles were performed with the annealing temperature at 55°C and a final extension of 72°C (10 min).

The amplified products were separated in a denaturing gradient gel of a 12% polyacrylamide gel [acrylamide-bisacrylamide (37.5 : 1, w/v)] containing a linear 30–60% denaturant gradient (100% denaturant corresponded to 7 M urea and 40% (v/v) formamide). DGGE was run for 18 h at 110 V on the DCode system (Bio-Rad, Hercules, CA, USA). The gel was stained with ethidium bromide and visualized by UV transillumination. Thereafter, the bands were excised from the DGGE gel and incubated in Milli-Q-water (30 *μ*L) for 24 h at 4°C. The eluent was then again amplified using the same PCR condition and primers but without GC-clamp.

### 2.5. Cloning and Sequencing

The amplified PCR products were purified using the QIAquick® Gel Extraction Kit (Qiagen, Hilden, Germany) and ligated into the pGEM-T Easy Vector (Promega, Madison, WI) according to the manufacturer's protocols. They were transformed into HIT*™* competent* Escherichia coli* DH5-*α* (RBC Bioscience, New Taipei, Taiwan) and plated on Luria-Bertani agar plates in the presence of ampicillin. The white colonies were selected and incubated in Luria-Bertani broth containing ampicillin (50 *μ*g/mL). DNA was purified using the QIAprep® Spin Mini-Prep Kit (Qiagen, Hilden, Germany). The purified products were sequenced by Solgent Inc. (Daejeon, South Korea), using an ABI 3730XL automatic DNA sequence (Carlsbad, CA, USA).

### 2.6. Statistical Analysis

All experiments were carried out in triplicate with standard deviation (SD) represented as bars wherever necessary using Microcal*™* Origin® Version 6.0. Effects of N, P, and B supplementation to the growth medium on total MCs production, Chl *a*, and MC content were statistically analyzed using multivariate analysis of variance (ANOVA). Data were explored using SPSS 16.0 software and log-transformed to give these an approximate normal distribution.

## 3. Results

### 3.1. Photoautotrophic Growth of the Target Strains in Different Nutrient Regimes (N, P, and B)

**Figure 1:**
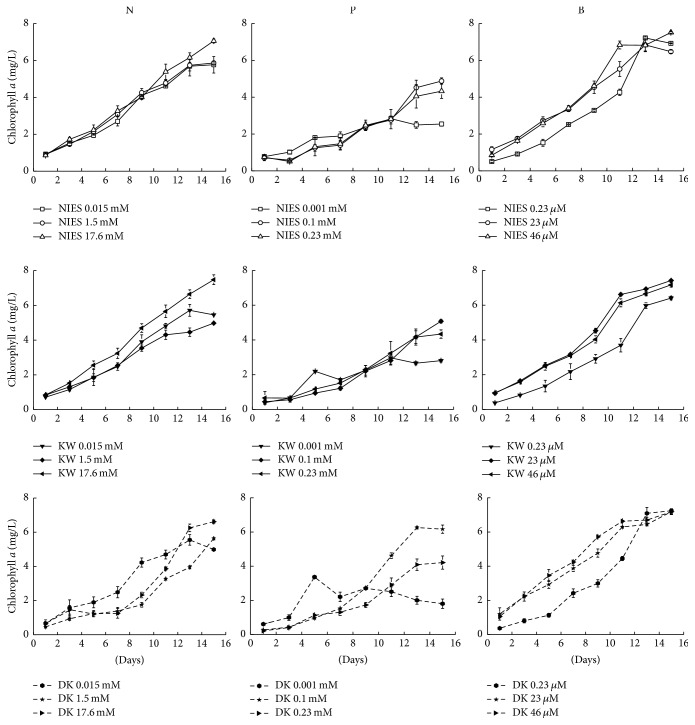
Growth curves of* M. aeruginosa* NIES 843, KW, and Durgakund (DK) strains under different concentrations of N, P, and B.

Photoautotrophic growth of target strains was monitored in low levels of nutrients (N, 0.015 mM; P, 0.001 mM; B, 0.23 *μ*M). These strains were transferred separately in medium having selected higher nutrient concentrations and growth behavior was monitored up to 15th d by measuring Chl *a* at periodic intervals (Figure 1). Chl *a* was significantly influenced by different concentrations of N (*F*_1,12_ = 120, *p* < 0.001), P (*F*_1,12_ = 5.4, *p* < 0.05), and B (*F*_1,12_ = 26.4, *p* < 0.001) in all three strains. Increased level of the N in the medium resulted in the higher Chl *a* yield (avg. 5 mg/L, 11th d) in the target strains. The yield was approximately 1.1 times higher in the cultures of all strains with 17.6 mM N. It was interesting that 100 times more concentration of N (1.5 mM) did not affect the Chl *a* yield (avg. 4.1 mg/L) in the target strains as compared to the growth in the lowest N concentration. The growth of Durgakund strain was lower as compared to NIES 843 and KW strains.

Likewise, the strains grown in varying levels of P behaved differently. The Chl *a* yield of the strains in low level of P (0.001 mM) was 1.67 times lower on an average basis as compared to the cultures with low level of N. Increase in the P concentration (100 times) in the growth medium increased the growth of the target strains by 1.21 times (avg. 3.4 mg/L) (Figure 1). The growth of Durgakund strain was better as compared to NIES 843 and KW strains. Decrease in the Chl *a* yield (1.1 times) of the target strains at 0.23 mM P indicated that there was no increase in the growth beyond a critical threshold level of P (0.1 mM). This was in contrast to the level of N in the cultures favoring the growth of the target strains. Interestingly, photoautotrophic growth of the strains was favored with increase in B levels (Figure 1). Average Chl *a* yield of the strains was 4.1, 6.2, and 6.5 mg/L in low (0.23 *μ*M) and higher concentrations (23 and 46 *μ*M), respectively.

### 3.2. Effect of Nutrient Concentrations on MC Variants

**Figure 2:**
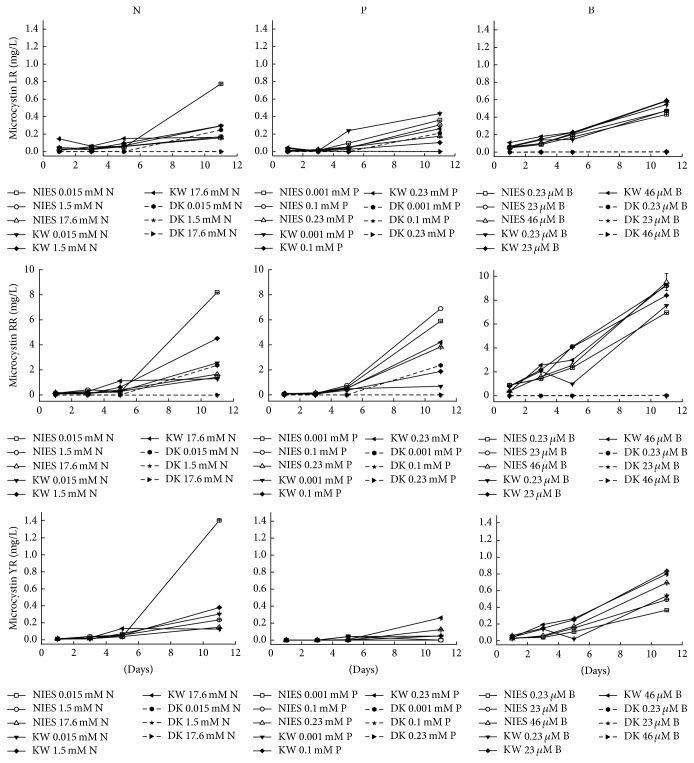
Variations in MC-LR, -RR, and -YR of* M. aeruginosa* NIES 843, KW, and Durgakund (DK) strains under different concentrations of N, P, and B.

Samples were collected simultaneously from the same set of cultures on 1, 3, 5, and 11 d for MC analysis. Intracellular and extracellular MCs were referred to as total MC for each variant. All three MC variants (MC-LR, -RR, and -YR) were detected in both NIES 843 and KW strains at different N concentrations with predominance of MC-RR (Figure 2). MC-YR was not detected in cultures of Durgakund strain at any of the N addition experiments. Total MC was significantly influenced by N (*F*_1,12_ = 653, *p* < 0.001), P (*F*_1,12_ = 1179, *p* < 0.001), and B (*F*_1,12_ = 1131, *p* < 0.001) in the target strains. MC content was also significantly influenced by N (*F*_1,12_ = 381, *p* < 0.001), P (*F*_1,12_ = 194, *p* < 0.001), and B (*F*_1,12_ = 718, *p* < 0.001). The highest levels of MC-LR (0.77 mg/L), -RR (8.2 mg/L), and -YR (1.4 mg/L) were recorded at 0.015 mM N in NIES 843. However, at higher concentrations of N (1.5 and 17.6 mM), the same levels of MC variants were recorded in both NIES 843 and KW strains (Figure 2). In comparison, Durgakund strain had the lowest concentration of MC-LR and -RR.

Likewise MC-YR was not detected in Durgakund strain growing in any of the P concentrations while higher level of MC-YR was recorded in KW strain at 0.23 mM P. Highest level of MC-LR and -RR was recorded in NIES 843 strain followed by KW strain and Durgakund strain (Figure 2). Higher level of P (0.23 mM) favored the production of MC-LR, -RR, and -YR in NIES 843. MC-YR was again not detected in Durgakund strain at any of the B incubated cultures. Higher B concentrations favored all MC variants in both NIES 843 and KW strains (Figure 2).

**Figure 3:**
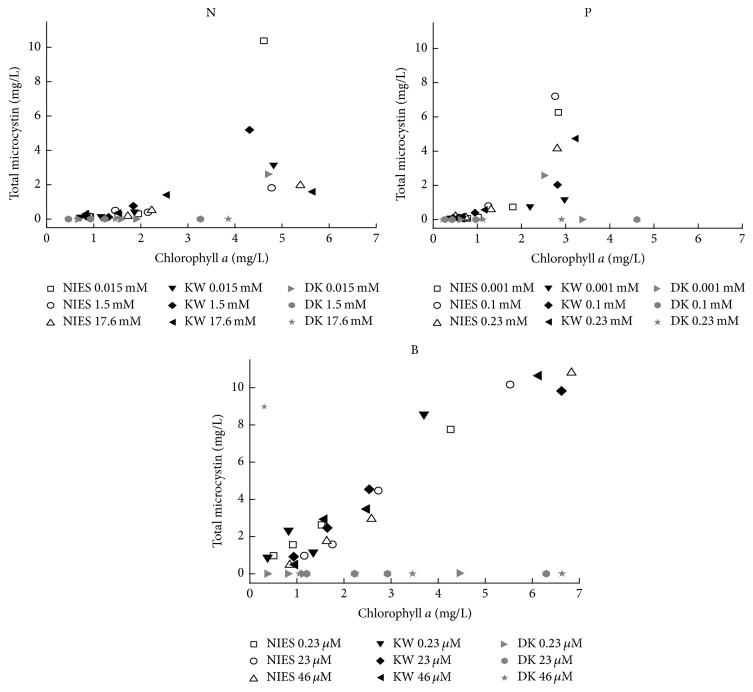
Total MC concentration as a function of chlorophyll *a* concentration in* M. aeruginosa* NIES 843, KW, and Durgakund (DK) strains under different concentrations of N, P, and B.

**Figure 4:**
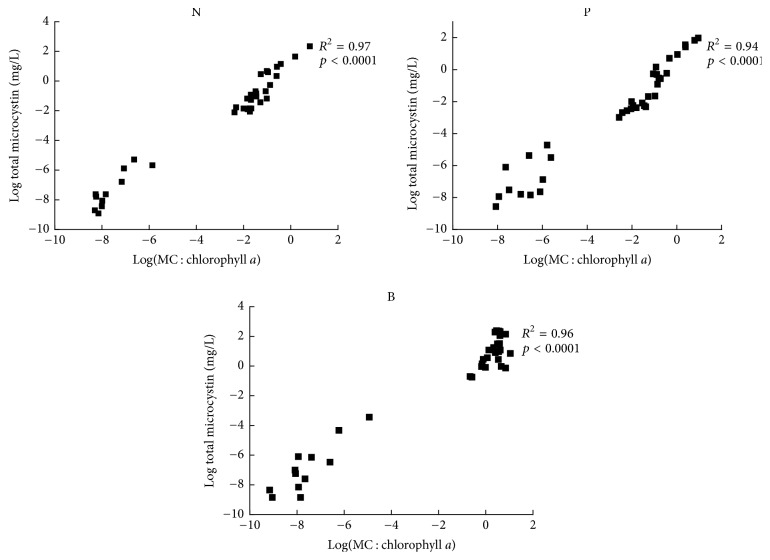
Relationship between MC content and total MC concentration in* M. aeruginosa* NIES 843, KW, and Durgakund (DK) strains under different concentrations of N, P, and B.

The total MC concentration in the cultures of the target strains linearly increased with the Chl *a* concentration (Figure 3). Very high total MC concentration was observed under different concentrations of B in NIES 843 and KW strains while total MC concentration was much lower in DK strain under various N, P, and B concentrations (Figure 3). Highly significant correlation was observed between MC concentration and total MC content under variable concentrations of N, P, and B in the target strains (Figure 4).

### 3.3. Effect of Nutrient Concentrations on* mcyA* Expression

**Figure 5:**
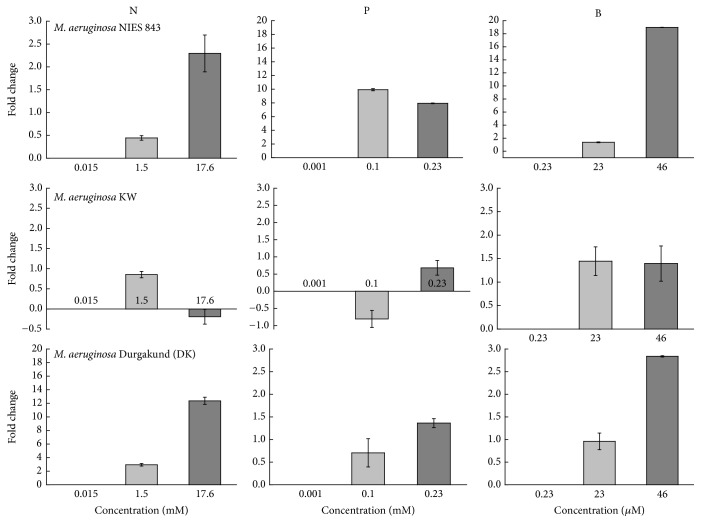
Effect of different concentrations of N, P, and B on* mcyA* expression of* M. aeruginosa* NIES 843,* M. aeruginosa* KW, and* Microcystis* Durgakund (DK) strain. The data were calculated relative to the expression in control cells (grown in low nutrient concentration) and normalized with the expression of the reference gene (16S rRNA).

Real-time RT-PCR was used to measure the expression of* mcyA* at different nutrient concentrations (N, P, and B) in the target strains (Figure 5).* mcyA* transcript increased at 1.5 and 17.6 mM N relative to the control (0.015 mM N). Maximum increase (12.4-fold) in* mcyA* transcript was recorded in Durgakund strain and only 2.3-fold increase in NIES 843.* mcyA* transcript in KW strain decreased (0.19-fold) relative to the control at 17.6 mM N. However,* mcyA* transcript increased in the target strains at 1.5 *μ*M N indicating a threshold level of N for optimum expression of* mcyA*. Similarly, increase in P levels (0.1 and 0.23 mM) increased the* mcyA* transcript in NIES 843 and Durgakund strains while only 0.23 mM P slightly increased the expression of* mcyA*. The maximum increase of* mcyA* transcript (10-fold) was recorded in NIES 843 at 0.1 mM P. In B-containing cultures, concentration-dependent increase in* mcyA* transcript was recorded in the target strains. The maximum increase (19-fold) in* mcyA* transcript was recorded in NIES 843 at 46 *μ*M B followed by Durgakund (2.8-fold) and KW strain (1.4-fold).

The correlation among growth, MC concentrations, MC content, and* mcyA* expression for the target strains is represented in Table 2. MC content per biomass increased with the total MC concentrations as significant correlation was observed among these (*p* < 0.0001, *R* ^2^ = 0.769). Chl *a* concentration and MC production were closely related in different concentrations of P in NIES 843 (*p* < 0.0001, *R* ^2^ = 0.835) and KW strains (*p* = 0.001, *R* ^2^ = 0.669) and of B in NIES 843 (*p* < 0.0001, *R* ^2^ = 0.960) and KW strains (*p* < 0.0001, *R* ^2^ = 0.918). Such relationship was rather weak in different concentrations of N in NIES 843 (*p* = 0.033, *R* ^2^ = 0.378) and KW strains (*p* = 0.004, *R* ^2^ = 0.584). In different nutrient regimes the expression of* mcyA* was not correlated with MC concentration (*p* = 0.222, *R* ^2^ = 0.342) and content (*p* = 0.333, *R* ^2^ = 0.232) in NIES 843. Similar trend was evident in Durgakund strain with MC concentration (*p* = 0.958, *R* ^2^ = 0.001) and content (*p* = 0.945, *R* ^2^ = 0.001). Contrary to this,* mcyA* expression pattern is directly reflected on MC concentration (*p* = 0.011, *R* ^2^ = 0.834) and content (*p* = 0.044, *R* ^2^ = 0.679) in KW strain.

### 3.4. Bacterial Community

**Figure 6:**
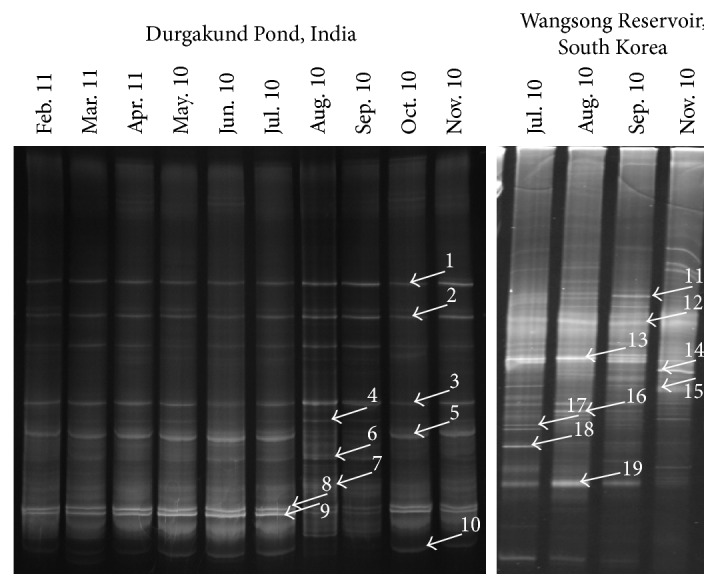
DGGE profile of 16S rDNA fragments of bacteria and cyanobacteria in bloom samples collected monthly from Durgakund Pond and Wangsong Reservoir. All labeled bands were excised from the gel, reamplified, and sequenced.

DGGE profile of water samples, collected during cyanobacterial bloom, from Durgakund Pond and Wangsong Reservoir for bacterial and cyanobacterial 16S rRNA gene is shown in Figure 6. All labeled bands were sequenced and most of the analyzed sequences showed 99% similarity to their closest relatives in the database (Table 3). Six sequences (bands 5, 10, 16, 17, 18, and 19) were related to Alphaproteobacteria and retrieved from all the samples except for August and September in Durgakund while bands 17 and 18 appeared in July and August in Wangsong Reservoir samples. Sequence related to Betaproteobacteria (band 4) appeared in August and September. One bacterial sequence (band 9) was related to Xanthomonadales order within Gammaproteobacteria and found in the Durgakund samples of all months. Among the cyanobacterial community, bands 8, 12, and 13 (intense) showed similarity to* M. aeruginosa* and were present in all the samples of Durgakund and Wangsong Reservoir. Interestingly, sequence (band 6) related to* M. wesenbergii* was more intense in August and September samples of Durgakund Pond. Sequence (band 2) related to* Merismopedia glauca* belonging to order Chroococcales was also seen in all the samples of Durgakund. In general, the banding pattern of DGGE in August and September samples differed from the samples of other months in Durgakund (Figure 6).

## 4. Discussion

Net production of MC was shown to be influenced by growth rate, that is, cell division process, reflecting a nearly linear correlation between them [33]. In the present study, the MC content per biomass increased with the total MC concentration but growth could not be correlated with total MC concentration in the target strains (Table 2). This was in tune with observations recorded earlier in the hepatotoxic* Microcystis* strains [15]. In this study, low N (0.015 mM) approximated eutrophic conditions in water bodies; therefore increased N concentration (maximum 17.6 mM) favored growth in the target strains. Higher level of N (1.5 mM and 17.6 mM) resulted in increased MC concentrations in the strains which is consistent with previous lab studies [34, 35]. Fluctuations in MC level of cyanobacteria with respect to N concentrations in medium have already been reported [16, 33, 36]. Recent study also demonstrated that N-limited conditions reduced the MC quota in* Microcystis aeruginosa* relative to nutrient-saturated conditions [37]. Increased MC content in higher levels of N seemed to be rational in this context; however composition and presence of MC variants also changed with strains. Composition and dominance of MC-RR variant in the present context could be explained on the basis that MC is an N-rich compound (over 14% of MC-LR on molecular weight basis) [38] and increased intracellular content of the N-rich amino acid arginine promotes production of the [Asp^3^] MC-RR variant in* M. aeruginosa* [35, 39].

The target strains showed better growth at 0.1 mM P, indicating it is a threshold limit.* M. aeruginosa* NIES 843 and KW followed the same trend in growth, while Durgakund strain seemed more sensitive to the changes in P concentrations. Fluctuations in MC levels of the target strains under varying P concentrations again indicate strain specific response to P. Interestingly, significant decrease in the total MC level was observed in Durgakund strain under higher P concentrations (0.1 and 0.23 mM). There are some reports where P limitation slightly reduced or did not influence the toxicity of* M. aeruginosa* [36, 37], while increase in MC production by* Microcystis* was also reported with decreasing concentrations of P in culture conditions [40, 41]. In contrast to these results, a positive correlation between MC content and increasing phosphate was observed in natural ecosystems suggesting that raised level of P increased the relative contribution of toxigenic cyanobacteria to total phytoplankton biomass [42, 43]. Several reports have also emphasized the importance of N : P ratios in MC production due to variations in response of* Microcystis* strains to N and P [14, 15, 44, 45]. For example, Lee et al. [44] reported higher MC and Chl *a* content at total N : P atomic ratios of 16 : 1 and 50 : 1. Furthermore, it was suggested that cellular MC content is a function of cellular nitrogen status and found to be strongly correlated with cellular protein content at N : P ratios between 18 and 51 in* M. aeruginosa* [34].

It is hard to devise a clear role of N and/or P in development of bloom and its toxicity; therefore we also investigated the effect of B, which has a potential role in signaling and interspecies communication. The role of B has also been shown in organization of cell walls, membrane function, metabolic activities, and stabilization of heterocyst in cyanobacteria [46]. Recently, a B-containing signal molecule (autoinducer AI-2), encoded by AI-2 synthase (*luxS*), has been reported by Chen et al. [20]. AI-2 is produced by a large number of bacterial species and proposed to serve as a universal signal for interspecies communication [47]. The production of signaling molecules called N-acyl homoserine-lactones (AHLs) was also reported for the first time in* Gloeothece* sp. [48] and* M. aeruginosa* [49]. They demonstrated that the concentration of the AHLs was cell density dependent and might play an important role in bloom formation. Since one of the potential roles of MCs in the producing organism was proposed in extracellular signaling, autoinduction, and maintenance of colonies [5, 6, 10], we studied the effect of B on MC production in* Microcystis* strains in the current study. It was interesting that B favored growth of all the target strains along with MC concentrations (Figure 3). The total concentrations of MC variants in Durgakund strain were lowest as compared to NIES 843 and KW strains under different concentrations of N, P, and B. This could be explained on the basis that the presence of toxic strains of cyanobacteria does not always indicate toxicity, as MC-producing cell quota may vary up to 3-4 orders of magnitude [50]. Since we have done lab-based study, cyanobacteria also undergo changes in loss of toxin production [51] and colonial morphology in* Microcystis*, if maintained in the culture for prolonged period [52].

Transcription of* mcy* genes in* M. aeruginosa* occurs* via* a central bidirectional promoter between* mcyA* and* mcyD*. A total of three NtcA (global nitrogen regulator) binding sites were identified in the* mcyA*/*D* promoter region, suggesting the role of nitrogen in controlling MC biosynthesis [53]. In this study, the overall patterns in* mcyA* expression were somewhat consistent with MC levels measured under different N concentrations in all the three strains but not directly correlated with the total MC concentrations (*p* = 0.273, *R* ^2^ = 0.287). This finding is consistent with observation where low N resulted in decrease in transcript levels of MC synthesizing genes [54]. However, Sevilla et al. [17] reported that the MC concentration in the cultures correlated with* mcyD* expression but the transcript level remained constant and was independent of nitrate availability. Recently, Horst et al. [37] suggested that MC quota was not driven by decrease in copy number of the* mcyB* gene but mediated by phenotypic responses of individual cells to N availability. As MC is made up of different modules, each having specific enzymatic function [4], N might be helpful in assembly of these modules explaining the increased MC concentration without any direct correlation with upregulation of transcription. An alternative explanation could be that there might be an initial accumulation of MC quota in the cells and since prokaryotic mRNA is highly unstable, these might have already been transcribed and translated leading to variable results at the time of expression analysis. However, further research on time dependent real-time analysis is needed.

In this study,* mcyA* transcript increased under higher P levels in the target strains. In another study, insignificant changes were observed in the relative quantification of* mcyD* in* M. aeruginosa* PCC 7806 under excess phosphate, while P-deficiency (N/P = 40 : 1) led to an increase in* mcyD* transcript and MC-LR per cell [55]. Increases in MC quotas and* mcyE* expression were also reported in Lake Rotorua with the increase in* Microcystis* cell concentrations [56]. They demonstrated that MC production was not always continuous and significant changes in* mcyE* expression occurred over smaller time frames.

Increase in* mcyA* expression in the target strains was consistent but not correlated with MCs levels under selected concentrations of B. Correlation analysis revealed that* mcyA* expression in only KW strain was directly linked with MC content (*p* = 0.044, *R* ^2^ = 0.679) and concentrations (*p* = 0.011, *R* ^2^ = 0.834) under different levels of N, P, and B. Increase in photoautotrophic growth and MC concentrations in the target strains suggested an indirect role of B but its role in nonheterocystous cyanobacteria is still not well documented. Furthermore, it was suggested that accumulation of* mcyB* in the lysing* Microcystis* cells stimulated the production of MCs in the remaining intact* Microcystis* cells [6]. Therefore, a more detailed study on direct role of B in signaling or MC production is needed particularly in nonnitrogen fixing species like* Microcystis*. Various field and laboratory studies failed to assign a single major factor responsible for promoting cyanobacterial bloom and its toxicity. This indicated a complex regulation of bloom development and its toxicity by biological and environmental variables. Induction of the growth of toxic* Microcystis* bloom is not yet known in spite of the fact that cyanobacterial blooms are regulated by various environmental factors. This study focused on response of* Microcystis* to differing nutrient concentrations on* mcy* transcription and MCs production which could help to decipher the possible regulation of the bloom growth and its toxicity and in timely management of the water bodies.

The interaction between bacteria and cyanobacteria is one of the biotic factors that influence the bloom dynamics [57]. Therefore, we also investigated the community compositional plasticity of heterotrophic bacteria associated with cyanobacterial blooms in Durgakund Pond and Wangsong Reservoir (Figure 6, Table 3). Bacterial groups, such as Alphaproteobacteria, Betaproteobacteria, and Gammaproteobacteria, were frequently found to be the dominant groups during the bloom of* Microcystis*. Earlier we have reported the dominance of* Microcystis* sp. in Durgakund Pond with the highest proportion of potentially toxigenic* Microcystis* sp. (14%) and MCs concentration in September [12]. Our observation was in tune with the findings that* Microcystis* bloom changed the microhabitat and quantity and quality of exudates and also microbes have the potential to control bloom development of potentially toxic cyanobacteria [9, 57]. However, in future a more comprehensive comparative study of bacteria associated with cyanobacteria and MC production in the field and lab cultures may provide insight in the complex regulatory mechanisms of MC production.

## 5. Conclusions

In summary, a comparison of photoautotrophic growth, MC content and concentration, and* mcyA* gene expression of three lab-grown* Microcystis* strains (NIES 843, KW, and Durgakund) in different nutrient regimes revealed that MC content per biomass and total MC concentration were highly correlated. MC production increased with increase in growth and this could have important implications for the management of freshwater bodies during* Microcystis* bloom upsurge. Lack of MC-YR in Durgakund strain indicated that MC concentration and variants were strain specific. Expression of* mcyA* showed nutrient concentration-dependent tendency but was not correlated with total MC concentration and therefore necessitates a time dependent real-time analysis for understanding the role of nutrients and other ecological variables in MC production. Natural associations of heterotrophic bacteria and cyanobacterial bloom were somewhat similar in freshwater bodies; however bacterial community showed variation during the different phases of the cyanobacterial bloom.

## Figures and Tables

**Table 1:** Primers used in this study.

Primers	Sequence	Description	Reference
*mcyA*-Cd 1F (forward)	5′-AAAATTAAAAGCCGTATCAAA-3′	*mcyA* (condensation domain)	[25]
*mcyA*-Cd 1R (reverse)	5′-AAAAGTGTTTTATTAGCGGCTCAT-3′		
GM5F-GC-clamp^a,b^	5′-GC-clamp-CCTACGGGAGGCAGCAG-3′	16S rRNA gene DGGE fragment amplification	[26]
786R (reverse)	5′-CTACCAGGGTATCTAATC-3′	16S rRNA amplification for real-time RT-PCR	[27]
27F (forward)	5′-AGAGTTTGATCMTGGCTCAG-3′	Complete sequence of 16S rRNA for nested-PCR	[28]
1525R (reverse)	5′-AAGGAGGTGATCCAGCC-3′	Complete sequence of 16S rRNA for nested-PCR	[28]
907R (reverse)	5′-CCGTCAATTCCTTTGAGTTT-3′	16S rRNA gene DGGE fragment amplification	[29]

^a^GC-clamp: 5′-CGCCCGCCGCGCGCGCCGCCCGCCCCGCCCCCGACGGGGGG-3′.

^b^GM5F (without GC-clamp) was used as forward primer for real-time RT-PCR.

**Table 2:** Coefficient of determination (*R* ^2^) among chlorophyll *a*, microcystin concentrations and content, and *mcyA* expression in *Microcystis* strains (NIES 843, KW, and Durgakund)^a^.

	Chlorophyll *a* (mg/L)	Total MC (mg/L)	MC content (mg/Chl *a*)	*mcyA* (fold change)
Chlorophyll *a*	1			
Total MC	0.110	1		
MC content	0.002	0.769^*∗∗∗*^	1	
*mcyA *	0.000	0.083	0.087	1

MC: microcystin; Chl *a*: chlorophyll *a*.

^a^Calculations based on data at 11th d, ^*∗∗∗*^ *p* < 0.0001.

**Table 3:** Phylogenetic affinity of *16S rRNA* gene sequences retrieved from monthly cyanobacterial bloom samples.

Band number	Name	Accession number	Similarity (%)	Taxonomic group
*Durgakund Pond*				
1	Uncultured bacterium	JQ906016	99	Uncultured bacterium
2	*Merismopedia glauca*	AJ781044	99	Chroococcales, Cyanobacteria
3	Uncultured bacterium^a^	KF418783	—	Uncultured bacterium
4	Uncultured beta-proteobacterium	JN371632	95	Betaproteobacteria
5	*Porphyrobacter* sp.	AB299749	99	Sphingomonadales, Alphaproteobacteria
6	*Microcystis wesenbergii*	AB666079	99	Chroococcales, Cyanobacteria
7	Uncultured bacterium	HQ653656	97	Uncultured bacterium
8	*Microcystis aeruginosa*	AF139304	99	Chroococcales, Cyanobacteria
9	*Aquimonas* sp.^a^	KF418784	—	Xanthomonadales, Gammaproteobacteria
10	*Roseomonas* sp.	HM124370	98	Rhodospirillales, Alphaproteobacteria

*Wangsong Reservoir*				
11	*Acinetobacter *sp.	KM108563	79	Pseudomonadales, Gammaproteobacteria
12	*Microcystis aeruginosa*	KF372572	98	Chroococcales, Cyanobacteria
13	*Microcystis aeruginosa*	KJ746519	100	Chroococcales, Cyanobacteria
14	*Malikia spinosa*	NR114228	99	Burkholderiales, Betaproteobacteria
15	*Aeromonas* sp.	KM363229	99	Aeromonadales, Gammaproteobacteria
16	*Rhodobacter* sp.	AB251408	93	Rhodobacterales, Alphaproteobacteria
17	Uncultured alpha-proteobacterium	HM153675	98	Alphaproteobacteria
18	*Roseomonas lacus*	JQ349047	99	Rhodospirillales, Alphaproteobacteria
19	Uncultured alpha-proteobacterium	HM153673	100	Alphaproteobacteria

^a^All the bands except 3 and 9 were based on published papers; therefore bands 3 and 9 were submitted to Genbank (BankIt1645319 Seq1 KF418783 for DGGE band #3; BankIt1646633 Seq1 KF418784 for DGGE band #9).

## References

[1] Carmichael W. W. (2001). Health effects of toxin-producing cyanobacteria: ‘The CyanoHABs’. *Human and Ecological Risk Assessment*.

[2] Welker M., Von Döhren H. (2006). Cyanobacterial peptides—nature's own combinatorial biosynthesis. *FEMS Microbiology Reviews*.

[3] Hudnell H. K., Dortch Q., Zenick H., Hudnell H. K. (2008). An overview of the interagency, international symposium on cyanobacterial harmful algal blooms (ISOC-HAB): advancing the scientific understanding of freshwater harmful algal blooms. *Cyanobacterial Harmful Algal Blooms: State of the Science and Research Needs*.

[4] Tillett D., Dittmann E., Erhard M., Von Döhren H., Börner T., Neilan B. A. (2000). Structural organization of microcystin biosynthesis in *Microcystis aeruginosa* PCC7806: an integrated peptide-polyketide synthetase system. *Chemistry and Biology*.

[5] Dittmann E., Erhard M., Kaebernick M. (2001). Altered expression of two light-dependent genes in a microcystin-lacking mutant of *Microcystis aeruginosa* PCC 7806. *Microbiology*.

[6] Schatz D., Keren Y., Vardi A. (2007). Towards clarification of the biological role of microcystins, a family of cyanobacterial toxins. *Environmental Microbiology*.

[7] Jähnichen S., Long B. M., Petzoldt T. (2011). Microcystin production by *Microcystis aeruginosa*: direct regulation by multiple environmental factors. *Harmful Algae*.

[8] Alexova R., Haynes P. A., Ferrari B. C., Neilan B. A. (2011). Comparative protein expression in different strains of the bloom-forming cyanobacterium *Microcystis aeruginosa*. *Molecular and Cellular Proteomics*.

[9] Dziallas C., Grossart H.-P. (2011). Increasing oxygen radicals and water temperature select for toxic *Microcystis* sp. *PLoS ONE*.

[10] Gan N., Xiao Y., Zhu L. (2012). The role of microcystins in maintaining colonies of bloom-forming *Microcystis* spp.. *Environmental Microbiology*.

[11] Lukač M., Aegerter R. (1993). Influence of trace metals on growth and toxin production of *Microcystis aeruginosa*. *Toxicon*.

[12] Srivastava A., Choi G.-G., Ahn C.-Y., Oh H.-M., Ravi A. K., Asthana R. K. (2012). Dynamics of microcystin production and quantification of potentially toxigenic *Microcystis* sp. using real-time PCR. *Water Research*.

[13] Rinta-Kanto J. M., Konopko E. A., DeBruyn J. M., Bourbonniere R. A., Boyer G. L., Wilhelm S. W. (2009). Lake Erie *Microcystis*: relationship between microcystin production, dynamics of genotypes and environmental parameters in a large lake. *Harmful Algae*.

[14] Paerl H. W., Gardner W. S., McCarthy M. J., Peierls B. L., Wilhelm S. W. (2014). Algal blooms: noteworthy nitrogen. *Science*.

[15] Vézie C., Rapala J., Vaitomaa J., Seitsonen J., Sivonen K. (2002). Effect of nitrogen and phosphorus on growth of toxic and nontoxic *Microcystis* strains and on intracellular microcystin concentrations. *Microbial Ecology*.

[16] Dai R., Liu H., Qu J., Zhao X., Ru J., Hou Y. (2008). Relationship of energy charge and toxin content of *Microcystis aeruginosa* in nitrogen-limited or phosphorous-limited cultures. *Toxicon*.

[17] Sevilla E., Martin-Luna B., Vela L., Teresa Bes M., Luisa Peleato M., Fillat M. F. (2010). Microcystin-LR synthesis as response to nitrogen: transcriptional analysis of the *mcyD* gene in *Microcystis aeruginosa* PCC7806. *Ecotoxicology*.

[18] Alexova R., Fujii M., Birch D. (2011). Iron uptake and toxin synthesis in the bloom-forming *Microcystis aeruginosa* under iron limitation. *Environmental Microbiology*.

[19] Kirkwood A. E., Nalewajko C., Fulthorpe R. R. (2006). The effects of cyanobacterial exudates on bacterial growth and biodegradation of organic contaminants. *Microbial Ecology*.

[20] Chen X., Schauder S., Potier N. (2002). Structural identification of a bacterial quorum-sensing signal containing boron. *Nature*.

[21] Rippka R., Deruelles J., Waterbury J. B., Herdman M., Stanier R. Y. (1979). Generic assignments, strain histories and properties of pure cultures of cyanobacteria. *Microbiology*.

[22] Shirai M., Ohotake A., Sano T. (1991). Toxicity and toxins of natural blooms and isolated strains of *Microcystis* spp. (Cyanobacteria) and improved procedure for purification of cultures. *Applied and Environmental Microbiology*.

[23] Redfield A. C., Daniel R. J. (1934). On the proportions of organic derivations in seawater and their relation to the composition of plankton. *James Johnstone Memorial Volume*.

[24] Myers J., Kratz W. A. (1955). Relation between pigment content and photosynthetic characteristics in a blue-green algae. *The Journal of General Physiology*.

[25] Hisbergues M., Christiansen G., Rouhiainen L., Sivonen K., Börner T. (2003). PCR-based identification of microcystin-producing genotypes of different cyanobacterial genera. *Archives of Microbiology*.

[26] Muyzer G., De Waal E. C., Uitterlinden A. G. (1993). Profiling of complex microbial populations by denaturing gradient gel electrophoresis analysis of polymerase chain reaction-amplified genes coding for 16S rRNA. *Applied and Environmental Microbiology*.

[27] Paster B. J., Dewhirst F. E., Olsen I., Fraser G. J. (1994). Phylogeny of *Bacteroides*, *Prevotella*, and *Porphyromonas* spp. and related bacteria. *Journal of Bacteriology*.

[28] Lane D. J., Stackebrandt E., Goodfellow M. (1991). *16S/23S rRNA* sequencing. *Nucleic Acid Techniques in Bacterial Systematics*.

[29] Teske A., Wawer C., Muyzer G., Ramsing N. B. (1996). Distribution of sulfate-reducing bacteria in a stratified fjord (Mariager Fjord, Denmark) as evaluated by most-probable-number counts and denaturing gradient gel electrophoresis of PCR-amplified ribosomal DNA fragments. *Applied and Environmental Microbiology*.

[30] Pfaffl M. W. (2001). A new mathematical model for relative quantification in real-time RT-PCR. *Nucleic Acids Research*.

[31] Sambrook J., Russell D. W. (2001). *Molecular Cloning, a Laboratory Manual*.

[32] Don R. H., Cox P. T., Wainwright B. J., Baker K., Mattick J. S. (1991). ‘Touchdown’ PCR to circumvent spurious priming during gene amplification. *Nucleic Acids Research*.

[33] Long B. M., Jones G. J., Orr P. T. (2001). Cellular microcystin content in N-limited *Microcystis aeruginosa* can be predicted from growth rate. *Applied and Environmental Microbiology*.

[34] Downing T. G., Sember C. S., Gehringer M. M., Leukes W. (2005). Medium N:P ratios and specific growth rate comodulate microcystin and protein content in *Microcystis aeruginosa* PCC7806 and *M. aeruginosa* UV027. *Microbial Ecology*.

[35] Van de Waal D. B., Verspagen J. M. H., Lürling M., Van Donk E., Visser P. M., Huisman J. (2009). The ecological stoichiometry of toxins produced by harmful cyanobacteria: an experimental test of the carbon-nutrient balance hypothesis. *Ecology Letters*.

[36] Codd G. A., Poon A. M., Rogers L. L., Gallon J. R. Cyanobacterial toxins.

[37] Horst G. P., Sarnelle O., White J. D., Hamilton S. K., Kaul R. B., Bressie J. D. (2014). Nitrogen availability increases the toxin quota of a harmful cyanobacterium, *Microcystis aeruginosa*. *Water Research*.

[38] Botes D. P., Wessels P. L., Kruger H. (1985). Structural studies on cyanoginosins-LR, -YR, -YA and -YM, peptide toxins from *Microcystis aeruginosa*. *Journal of the Chemical Society, Perkin Transactions*.

[39] Van de Waal D. B., Ferreruela G., Tonk L. (2010). Pulsed nitrogen supply induces dynamic changes in the amino acid compositionand microcystin production of the harmful cyanobacterium *Planktothrix agardhii*. *FEMS Microbiology Ecology*.

[40] Oh H.-M., Lee S. J., Jang M.-H., Yoon B.-D. (2000). Microcystin production by *Microcystis aeruginosa* in a phosphorus-limited chemostat. *Applied and Environmental Microbiology*.

[41] Kameyama K., Sugiura N., Isoda H., Inamori Y., Maekawa T. (2002). Effect of nitrate and phosphate concentration on production of microcystins by *Microcystis viridis* NIES 102. *Aquatic Ecosystem Health and Management*.

[42] Giani A., Bird D. F., Prairie Y. T., Lawrence J. F. (2005). Empirical study of cyanobacterial toxicity along a trophic gradient of lakes. *Canadian Journal of Fisheries and Aquatic Sciences*.

[43] Izydorczyk K., Jurczak T., Wojtal-Frankiewicz A., Skowron A., Mankiewicz-Boczek J., Tarczyńska M. (2008). Influence of abiotic and biotic factors on microcystin content in *Microcystis aeruginosa* cells in a eutrophic temperate reservoir. *Journal of Plankton Research*.

[44] Lee S. J., Jang M.-H., Kim H.-S., Yoon B.-D., Oh H.-M. (2000). Variation of microcystin content of *Microcystis aeruginosa* relative to medium N:P ratio and growth stage. *Journal of Applied Microbiology*.

[45] Lewis W. M., Wurtsbaugh W. A., Paerl H. W. (2011). Rationale for control of anthropogenic nitrogen and phosphorus to reduce eutrophication of inland waters. *Environmental Science and Technology*.

[46] Brown P. H., Bellaloui N., Wimmer M. A. (2002). Boron in plant biology. *Plant Biology*.

[47] Surette M. G., Miller M. B., Bassler B. L. (1999). Quorum sensing in *Escherichia coli*, *Salmonella typhimurium*, and *Vibrio harveyi*: a new family of genes responsible for autoinducer production. *Proceedings of the National Academy of Sciences of the United States of America*.

[48] Sharif D. I., Gallon J., Smith C. J., Dudley E. D. (2008). Quorum sensing in *Cyanobacteria*: *N*-octanoyl-homoserine lactone release and response, by the epilithic colonial cyanobacterium *Gloeothece* PCC6909. *ISME Journal*.

[49] Zhai C., Zhang P., Shen F., Zhou C., Liu C. (2012). Does *Microcystis aeruginosa* have quorum sensing?. *FEMS Microbiology Letters*.

[50] Blackburn S. I., Bolch C. J. S., Jones G. J., Negri A. P., Orr P. T., Davis J. R. D. (1997). Cyanobacterial blooms: why are they toxic?. *Managing Algal Blooms: Outcomes from the CSIRO Blue Green Algal Research Program*.

[51] Schatz D., Keren Y., Hadas O., Carmeli S., Sukenik A., Kaplan A. (2005). Ecological implications of the emergence of non-toxic subcultures from toxic *Microcystis* strains. *Environmental Microbiology*.

[52] Zhang M., Kong F., Tan X., Yang Z., Cao H., Xing P. (2007). Biochemical, morphological, and genetic variations in *Microcystis aeruginosa* due to colony disaggregation. *World Journal of Microbiology and Biotechnology*.

[53] Ginn H. P., Pearson L. A., Neilan B. A. (2010). NtcA from *Microcystis aeruginosa* PCC 7806 is autoregulatory and binds to the microcystin promoter. *Applied and Environmental Microbiology*.

[54] Harke M. J., Gobler C. J. (2013). Global transcriptional responses of the toxic cyanobacterium, *Microcystis aeruginosa*, to nitrogen stress, phosphorus stress, and growth on organic matter. *PLoS ONE*.

[55] Kuniyoshi T. M., Sevilla E., Bes M. T., Fillat M. F., Peleato M. L. (2013). Phosphate deficiency (N/P 40:1) induces *mcyD* transcription and microcystin synthesis in *Microcystis aeruginosa* PCC7806. *Plant Physiology and Biochemistry*.

[56] Wood S. A., Rueckert A., Hamilton D. P., Cary S. C., Dietrich D. R. (2011). Switching toxin production on and off: intermittent microcystin synthesis in a *Microcystis* bloom. *Environmental Microbiology Reports*.

[57] Dziallas C., Grossart H.-P. (2011). Temperature and biotic factors influence bacterial communities associated with the cyanobacterium *Microcystis* sp. *Environmental Microbiology*.

